# Octopus Watch Fosters Family Resilience by Enhancing Occupational Engagement for Children with Spina Bifida and/or Hydrocephalus: Pilot Study

**DOI:** 10.3390/ijerph17228316

**Published:** 2020-11-10

**Authors:** Mark Jennings, Aoife Guilfoyle, James Green, Yvonne Cleary, Rosemary Joan Gowran

**Affiliations:** 1 Discipline Occupational Therapy, School of Allied Health, Faculty of Education and Health Sciences, University of Limerick, V94 T9PX Limerick, Ireland; markjennings200@gmail.com (M.J.); guilfoyle.aoife@gmail.com (A.G.); 2 School of Allied Health, Faculty of Education and Health Sciences, Physical Activity for Health (PAfH), Health Research Institute, University of Limerick, V94 T9PX Limerick, Ireland; James.Green@ul.ie; 3 Technical Communication and Instructional Design, University of Limerick, V94 T9PX Limerick, Ireland; Yvonne.Cleary@ul.ie; 4 Discipline Occupational Therapy, School of Allied Health, Faculty of Education and Health Sciences, Health Research Institute, Health Implementation Science and Technology (HIST), University of Limerick, V94 T9PX Limerick, Ireland; 5 School of Health and Sports Science, University of the Sunshine Coast, Maroochydore DC QLD 4558, Australia; 6 Assisting Living and Learning (ALL), Institute Maynooth University, Maynooth, W23 VP22 Co. Kildare, Ireland

**Keywords:** occupational engagement, spina bifida and/or hydrocephalus, sustainable assistive technology, family resilience

## Abstract

Background: Children with spina bifida and/or hydrocephalus (SB&/H) often experience difficulties with activities of daily living (ADLs) due to impaired executive functioning, increasing sedentary behaviours. The HeyJoy Octopus watch, a child-friendly icon-based smartwatch could be used as an enabler to promote purposeful ADLs (i.e., goal-orientated ADLs). Objective: to investigate the effectiveness of the Octopus watch in promoting purposeful ADLs for children living with SB&/H (<8 years). Methods: Mixed-methods engaging parents and children in four phases: (1) Administered demographic questionnaire, semi-structured interview, childhood executive functioning inventory (CHEXI) and the Canadian occupational performance measure (COPM); focus group one introducing the study, information pack using smartwatch and photovoice data collection methods. (2) Measured baseline movement for four days with smartwatch without using functions. (3) Measured activity for 16-days while using the smartwatch. (4) Re-administered assessments and conducted a second focus group based on photovoice narratives. Results: movement data recorded for four participants, three of four showed mean activity increase (36%). N-of-1 analyses found one participant showed clear improvement (*p* = 0.021, *r*^2^ = 0.28). Mean inhibition decreased by 16.4%, and mean change in COPM performance and satisfaction scores were 2.1 and 2.4, respectively. The photovoice narrative focus group supports findings evidenced with improved daily routines. Conclusions: The Octopus watch is an innovative early intervention that can promote purposeful ADLs, fostering family resilience by enhancing occupational engagement. Further research is required.

## 1. Introduction

Spina bifida (SB) is a congenital deformity of the neural tube and is the most common neural tube defect (NTDs) in children [1]. In Ireland, NTDs are amongst the highest incidence in the world with 1.17 per 1000 births [2]. SB is caused by the unfinished closure of the neural tube and leads to protrusion of nerves and spinal membranes during the early days of gestation [3]. This unfinished closure is associated with several abnormalities in the brain, including Arnold–Chiari malformation (Chiari II) and hydrocephalous [4]. Specifically, hydrocephalus occurs in 70–90% of individuals living with SB [5]. Hydrocephalus can occur without SB and is attributed to a disorder of cerebrospinal fluid (CFS), which abnormally expands the cerebral ventricles leading to increased intracranial pressure [6], often requiring CSF shunting to decrease this pressure [7]. Subsequently, individuals living with spina bifida and/or hydrocephalous (SB&/H) experience pervasive impacts, including: motor and sensory impairments, disturbances with bladder and bowel incontinence, and reduced cognitive functioning [8,9]. These all negatively impact independence in activities of daily living (ADLs).

Strömfors et al. identified three significant barriers to independence in ADLs, including motivation, preparedness and planning [10]. These perceived barriers are evident across SB&/H literature and could be directly linked to impaired executive functioning (EF), a core cognitive deficit identified in the SB&/H cohort [11,12]. Impaired EF impedes goal-directed behaviours when adapting to altered circumstances [13], hinders independent living skills [14,15], increases sedentary behaviours [16] and heightens prevalence of secondary conditions [17,18]. Difficulties associated with goal-directed behaviours can result from reduced alertness, a fundamental aspect of attention [19]. Despite the documented concern of impaired EF, limited studies address intervention strategies for individuals living with SB&/H [12]. Therefore, a healthy behaviour intervention is necessary.

In enhancing health behaviours for individuals living with SB&/H, physical activity (PA) is an important aspect to consider [20]. PA interventions for chronic conditions are well supported in the literature, with PA recognised as a leading indicator of good health, decreased morbidity and mortality [21]. However, PA interventions generally focus on objective PA (e.g., walking on a treadmill), thus, sustainability is questionable for the SB&/H cohort due to associated difficulties with goal-directed behaviours [22]. Bloemen et al. explored environmental and personal factors influencing participation in PA, with findings indicating that individualised interventions are more likely to improve participation [3]. Accordingly, a behaviour promoting intervention focusing on ADLs tailored to meet the individual needs may increase PA.

Researchers are increasingly recognising the significance of self-management interventions in supporting living with chronic illness (including SB&/H) to enhance independence with ADLs and ultimately improve health outcomes [17]. Systematic reviews found that self-management interventions among adults with chronic illness improved health outcomes, self-efficacy, quality of life and reduced morbidity [23,24]. However, a discernible gap is evident regarding behaviour promoting interventions for the SB&/H cohort, with most interventions focused on adults [25] and lacking synthesis regarding children and young people [26]. Moreover, significant attention must be given to children (<8 years) to promote self-management skills (ADLs), as children encounter several challenging developmental transitions [27,28]. For instance, motor developmental millstones in SB children (see Table 1) usually develop later than typically developing children [29].

In recent years, wearable assistive technology such as PA trackers has grown tremendously in popularity [30,31]. Healthcare providers and clients anticipate a role for wearable PA trackers in improving health, satisfaction and quality of care [32] with new technologies such as smartphone apps facilitating ADL interventions for individuals living with chronic conditions and disabilities [33,34]. For example, Dicianno et al. found significant improvements in self-management skills with a mobile health application for individuals living with SB [35]. However, assistive technology abandonment is prevalent with many assistive technologies not suitable for the SB&/H paediatric population, due to technology that is not child friendly, and the lack of a key purposeful ADL element, ‘play’ [36]. Children want to explore the environment in a playful manner [36]. Play is a critical part of a child’s life and contributes to the development of motor, sensory and cognitive processes [37].

Accordingly, an intervention that promotes a play element into ADLs is both a meaningful and purposeful activity for a child [38]. Thus, a plausible early intervention to help facilitate and promote purposeful ADLs could be the novel HeyJoy Octopus watch [39]. The manufacturer claims their product is the first child-friendly icon-based smartwatch manufactured to promote purposeful ADLs [39]. The element of ‘play’ is implemented into the watch with a reward system; for example, when a child completes an ADL the child is rewarded with three full stars out of three displaying on the watch screen, incorporating components of both joy and fun [39,40]. Furthermore, this pilot study sought to evaluate the benefits and sustainability of the Octopus watch use in partnership with Spina Bifida Hydrocephalus Ireland (SBHI), assessing its effectiveness and potential for provision to families nationally.

Therefore, the primary objective of this pilot study is to address whether the Octopus watch is a feasible intervention to promote purposeful ADLs in a group of children (<8 years) living with SB&/H. Thus, the study aims were: (1) to investigate the potential of the watch to increase PA; (2) to examine whether the watch compensates for impaired EF; (3) to explore the potential effects of the intervention on purposeful ADLs, and (4) to explore user experience and technology acceptance of the Octopus watch.

## 2. Methods

### 2.1. Pilot Research Design

Quantitative pretest-posttest and repeated measure single-case n-of-1 designs were used, combined with photovoice methodology to supplement the findings [41]. Photovoice enables participants to tell their story through photographs and is a powerful communicator for children [42,43,44]. Single-case or n-of-1 designs allow test effects or relationships at the individual level, allowing inference to be derived per participant [45,46].

### 2.2. Participants

Participants were enrolled if they met the inclusion criteria: (1) a child under eight years old who has SB&/H and is supported by a parent or guardian, (2) has English as their first language, (3) meets the minimum requirement of functional ability to self-propel a wheelchair (see Table 2). Participants were excluded if they did not meet any of the above criteria.

Five families (parents/guardians of children living with SB&/H) were recruited through the gatekeeper (SBHI) via convenience sampling, with four families (80%) completing the full study (see Table 3). Written consent and assent were obtained from all children and parents before participating in the study. Pseudonyms were used to protect the participants’ confidentiality. Approval was obtained from the Education and Health Sciences Research Ethics Committee (2019_05_23_EHS).

### 2.3. Equipment

#### HeyJoy Octopus Watch v2

**Figure 1:**
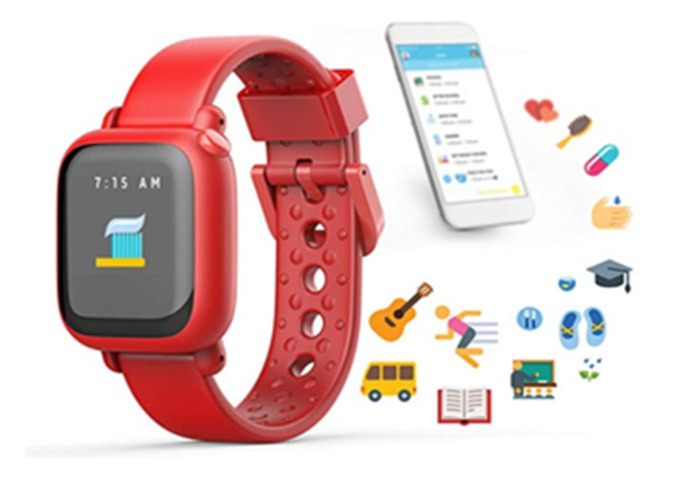
Equipment used: the HeyJoy Octopus watch.

The Octopus watch developed by HeyJoy is designed to empower children by teaching the concept of time and routine, while also encouraging them to stay active with a built-in fitness tracker [39]. The watch links time to activities through visual icons, e.g., when it is time to brush teeth, a toothbrush icon appears on the watch screen (see Figure 1). Over 2000 ADL icons are available on the accompanying smartphone application, including in the following areas: self-care, playtime, house chores, and mealtimes. The smartphone application is used to manage and monitor ADLs.

### 2.4. Procedure

The study commenced over four phases: pre-test, baseline, intervention and post-test.

Phase one: the pre-test, participants completed a demographic survey (Supplementary Materials), the childhood executive functioning inventory (CHEXI), a semi-structured interview (Supplementary Materials), and the Canadian occupational performance measure (COPM). During this phase, the participants were given the Octopus watch and information pack, with a supplementary PowerPoint presentation to guide a focus group. Focus groups provide a sense of security for participants and allow for ease of conversation among peers [49,50]. The focus group discussion centred around one key question for the participants: “what are your expectations (hopes and/or concerns) for the Octopus watch?” with the answers to this question intended to form a reference point for discussion in the follow-up session. Following the advice of Wang and Burris, the initial focus group also introduced the concept and methodology of photovoice [51].

Phase two: Comprised of collecting baseline activity while carrying out normal day-to-day routines without using Octopus-watch icons/schedule functions. Trost et al. recommend that four days or more is considered adequate for gathering accurate reliability of accelerometer data, providing the rationale for a four-day baseline measurement [52].

Phase three: Consisted of two parts (1) the intervention and measuring activity for 16-days while using the icon functions on the Octopus watch during the daily routine (see Supplementary Materials for Octopus watch instructions). (2) Participants took a minimum of five photographs on their own smartphones “that best depict your experiences of using the Octopus watch.” The mobile application ‘WhatsApp’ was used to transfer photographs to the research team. WhatsApp has end-to-end encryption that facilitates secure personal data transfer [53].

Phase four: The post-test was completed with the parents using the CHEXI, COPM and a short semi-structured interview (Supplementary Materials). Additionally, the baseline and intervention PA data were extracted from the mobile phone app and imputed into an Excel file. The participants’ chosen photographs were included in the PowerPoint presentation and displayed at the second focus group. The second focus group involved each participant explaining and discussing their photographs within a group setting. Written photograph narrations were collected by the researchers.

### 2.5. Measures

#### 2.5.1. Demographic Survey

Parents filled out demographic information (see Supplementary Materials), adapted from Vanderbom’s study [54].

#### 2.5.2. Short Semi-Structured Interviews

The pre-test interview explored barriers to ADLs and better informed the COPM assessment [55]. The post-test interview explored the effectiveness of the Octopus watch. Both interview questions used were semi-standardised, with parent perspectives taken during the interviews.

#### 2.5.3. Childhood Executive Functioning Inventory (CHEXI)

The CHEXI is a 24-item inventory of EF for children [56]. The two primary domains are working memory (working memory and planning sub-scales) and inhibition (regulation and inhibition sub-scales). The CHEXI displays acceptable levels of test-retest reliability (*r* > 0.74) and internal consistency (Cronbach’s α > 0.85) using parent scoring approximately three weeks apart [57]. Parents use the five-point Likert scale to rate each item from 1 = ‘definitely not true’ to 5 = ‘definitely true’, with EF difficulties indicated by higher scores [58].

#### 2.5.4. Canadian Occupational Performance Measure (COPM)

The COPM is a client-centred semi-structured interview that measures the self-perceived evaluation of occupational performance (OP) and occupational satisfaction (OS) domains for three activity categories: productivity, self-care and leisure [59]. For this study, the parent identified their child’s most purposeful activities that are difficult to perform, and rated these on a 10-point Likert-type scale. The COPM is considered a satisfactory measure of OP [55] and has an interclass correlation coefficient ranging from 0.73–0.93 [60].

#### 2.5.5. Activity Measure (HeyJoy Octopus Watch v2)

The proprietor describes the smartwatch activity monitoring as using 3-axis digital accelerometry and a digital gyroscope. Proprietary technologies are used to compute a measure of activity, known as ‘octo-points’ which we used as a proxy for PA. 6000 ‘octo-points’ equate to about 60 min of activity [39]. Thus, the octo-points provide an adequate measure for demonstrating the change in ADL activity.

### 2.6. Quantitative Analyses

Quantitative CHEXI and COPM data were analysed using descriptive statistics, including means and standard deviations, completed using Excel formulas on a Microsoft Excel spreadsheet. Paired samples t-tests were performed to investigate changes. All statistical analysis was conducted on IBM SPSS.26 using a 5% level of significance.

The PA time series data were plotted and visually examined to explore changes in activity during baseline and intervention phases for each participant. Although visual inspection of this data is recommended [61], recent studies show conventional statistics can overestimate intervention effects due to autocorrelation of time series data [62,63]. Autocorrelation is the possibility that on any given day, the participant’s PA output may be correlated with later or previous days, caused by data points being collected relatively close in time [45]. For example, tiredness today is partially predicted by tiredness yesterday. Therefore, each data set was analysed for autocorrelation. Some participants showed clear evidence of autocorrelations and in this case, a conservative response was to use a pre-whitening method for all participants [46], to satisfy the assumption that each single data point is independent [64]. The pre-whitened PA (octo-points) outcome was analysed with a linear regression using dummy coding (0 = baseline, 1 = intervention). Cohen effect size guidelines were used for interpretation, *r*^2^ ≥ 0.25, *r*^2^ ≥ 0.09, *r*^2^ ≥ 0.01 representing large, medium and small effect sizes, respectively [65].

Across all PA outputs, 10 random data points of 80 were missing (12.5%), with the majority recording zero octo-points (*n* = 9), due to the accelerometer data not recording any PA. A further data point was deemed missing as a parent violated the experimental protocol by wearing the watch. Missing data points were imputed using a simple averaging approach either side of the missing data [46].

### 2.7. Qualitative Analyses

#### 2.7.1. Focus Groups

An ethnographic approach was employed with each participant initially discussed as a case study outlining the content of photographs provided. An ethnographic approach is relevant to this research due to multiple data sources, small sample size and ethical considerations [66]. The data were recorded and transcribed verbatim. The principal researchers read and re-read the transcripts to become accustomed to the data. Thematic analysis techniques described by Braun and Clarke [67] were used with NVivo software [68]. Focus group transcripts and photograph narratives were analysed initially by identifying common themes. Nodes representing each theme were created in NVivo and data were reviewed to determine specific quotations relating to each theme. Photographs were subsequently selected to determine the best representation of themes.

#### 2.7.2. Semi-Structured Interviews

Content analyses were employed in conformity with the Graneheim and Lundman recommendations for both pre and post individual semi-structured interviews (details attached in supplementary file) [69]. No statistical software was used, and no pre-existing themes or criteria were identified. Transcripts were independently coded and cross-checked to ensure credibility of emergent themes [70].

## 3. Results

Four of the five participants completed the study, and one participant withdrew due to illness that compromised their ability to participate during the timeframe of the study. Ages ranged from 3 to 7 years with an equal ratio of males to females (2:2). Three of the four participants used assistive devices for mobility (i.e., wheelchair) with one child independently ambulant and one child independently ambulant for short distances. All participants attended the initial focus group with two of the four participants attending the second focus group, with individual feedback provided by participants unable to attend.

### 3.1. Quantitative Results

Based on the primary objective to assess the feasibility of the Octopus watch as an early-intervention, findings are presented with quantitative results first, followed by supporting photovoice narrative themes.

#### 3.1.1. Physical Activity

**Figure 2:**
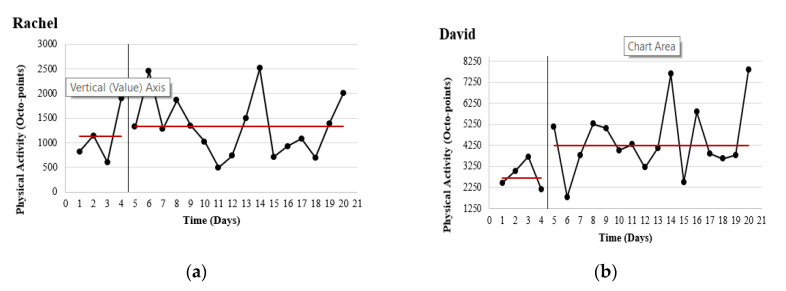

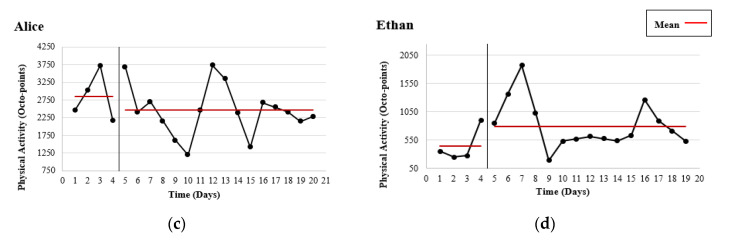
Graphical depiction of physical activity (PA) at baseline (Days 1–4) and during the Octopus watch intervention (Days 5–20) for each participant.

Research aim 1. To investigate the watch’s potential in promoting PA, visual plots were used to display the mean change in PA outputs (octo-points) from baseline to intervention (see Figure 2). Overall, the participants showed a mean activity increase of 36% (1865 octo-points). Rachel increased by 18.4% (207 octo-points). David increased by 58% (1563.6 octo-points). Alice decreased by −12.4% (355 octo-points). However, the vibration prompt function on the watch did not work correctly for this participant. Finally, Ethan increased by 78.1% (347.6 octo-points).

A corresponding n-of-1 analysis for each participant on the pre-whitened data [45,46] is presented in Table 4. Though descriptive statistics for PA appeared promising for three of the four participants, only David had a statistically significant change from baseline to intervention (*p* = 0.021) with a large effect size (*r*^2^ = 0.28). Changes recorded for Rachel, Alice and Ethan were not statistically significant.

#### 3.1.2. CHEXI

Research aim 2. To examine if the watch compensates for impaired EF (see Table 5). There was minimal change in the working memory domain pre to post-intervention (−0.9%). However, there were more promising decreases in the inhibition domain (−16.4%).

#### 3.1.3. COPM

Research aim 3. To explore the potential effects of the intervention on purposeful ADLs, every participant created five user-centred goals based on the COPM assessment (see Table 6). Overall, the mean quantitative performance and satisfaction scores on the COPM increased from pre-test to post-test by 2.1 and 2.4, respectively.

### 3.2. Qualitative Findings

Research aim 4. To explore user experience and technology acceptance of the watch. The semi-structured interviews, qualitative COPM components (see content analysis in Supplementary Materials) and photovoice narrative themes provide a more comprehensive and holistic view of the quantitative findings, illuminating factors that influenced the COPM, PA and EF outcomes. These are advantages of analysing qualitative data in mixed-method studies [71].

#### 3.2.1. Rachel

Rachel attended both sessions with her mother, Claire, who took and provided the photographs of Rachel’s experience with her Octopus watch. During the initial focus group, Claire expressed her hope for Rachel to become more independent as a result of wearing the Octopus watch. She explained that Rachel is often slower than her siblings at eating breakfast and getting ready for school. She was quite eager for Rachel to begin to take responsibility for initiating tasks.

*“For her…to say I want to brush my teeth or to wash my hands, you know to get her feeding as well, to finish her breakfast with the rest of us, you know, that kind of a way. For her to become more independent”.*

**Figure 3:**
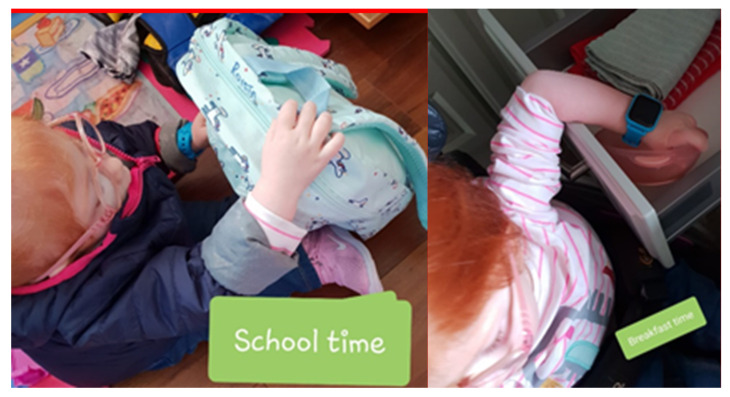
Rachel getting ready for school in the morning.

Claire provided five photographs. Claire captioned the photographs and provided narratives. Captions were as follows; ‘school time’, ‘breakfast time’, ‘storytime’, ‘playing outside’ and ‘time to wash hands’. Claire explained that she had chosen these specific photographs as they represented successes for Rachel. The Octopus watch supported Rachel’s independence and autonomous initiation of tasks. In the mornings, she helped her mother by bringing her breakfast bowl from the cupboard and having her schoolbag ready for school. Rachel also now independently chooses her storybook before bed without being prompted by her mother (see Figure 3).

#### 3.2.2. David

David attended the initial focus group with his mother, Ellen. Ellen attended the second quantitative session but was unable to participate in the second focus group. However, she completed an individual session, discussing both the provided photographs and the family’s experience of the watch. Ellen explained that the family had previously attempted to establish a morning routine with David by using various aids and strategies, but without success. At the initial focus group, she explained that her greatest expectation for the Octopus watch would be for it to help the whole family by assisting David with achieving tasks in the morning.

*“I suppose I hope that our morning routine might get a bit better, definitely, and you know, that we’re not just going to have battles every morning trying to get things achieved, especially with other kids in the house…it’s just mayhem in the mornings”.*

**Figure 4:**
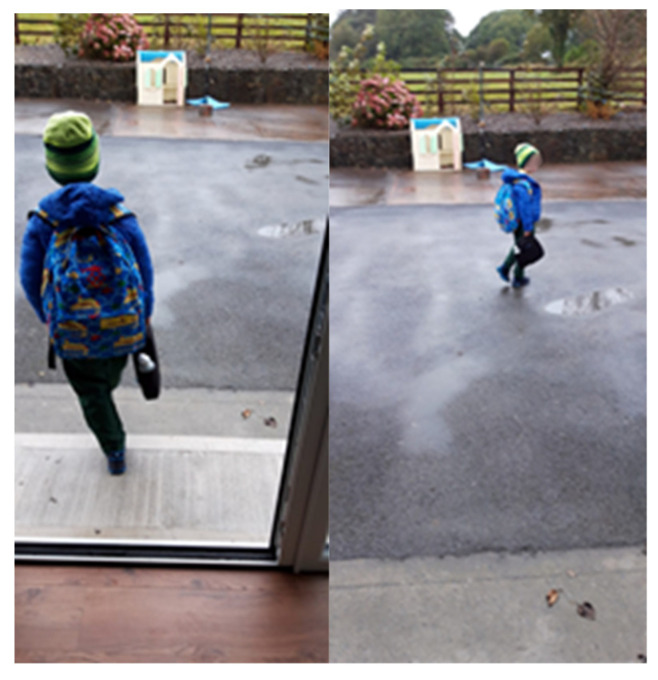
David on his way to school.

David’s family provided seven photographs. Ellen engaged in individual discussion with comments noted. Of the seven photographs provided, six depict aspects of David’s morning routine having a positive influence. These activities include: eating breakfast, brushing teeth, combing hair, and being on-time for school. Ellen explained that the Octopus watch had a profound impact on establishing a morning routine for David and now he no longer relies on prompts from the watch to complete tasks. In Figure 4, Ellen explains that David is now much better at leaving for school on time. Ellen also noted that David has made huge progress with his night-time routine.

#### 3.2.3. Alice

Alice attended the initial focus group with both her parents, and her mother, Anna, participated in the second session alone. Alice’s parents hoped the watch would assist Alice to become more independent and take responsibility for initiating tasks, especially in the morning as “mornings are generally hectic times”.

**Figure 5:**
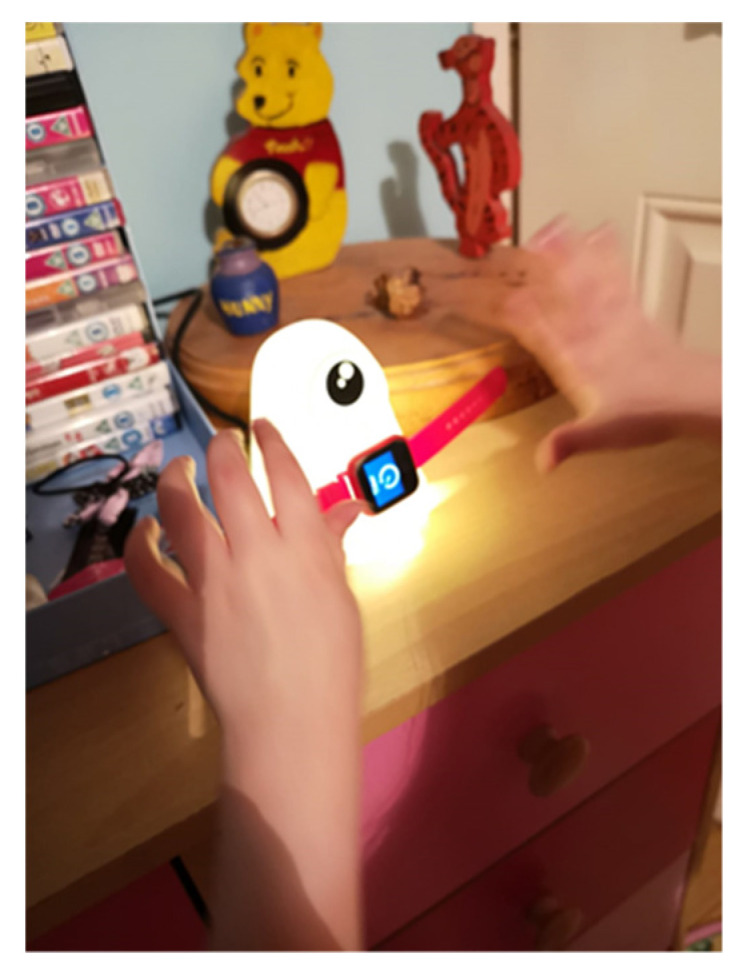
Alice ensures to place her watch on the charging port each night.

Alice’s family provided five photographs depicting their experiences of using the Octopus watch and discussed these photographs at the second focus group. These photographs included: going shopping, free time, play-school, charging the watch, and enabling the vibrate function. The greatest success experienced by Alice was her increased socialization associated with wearing the Octopus watch (see Figure 5) and increased autonomy with ensuring that the watch was charged each night.

#### 3.2.4. Ethan

**Figure 6:**
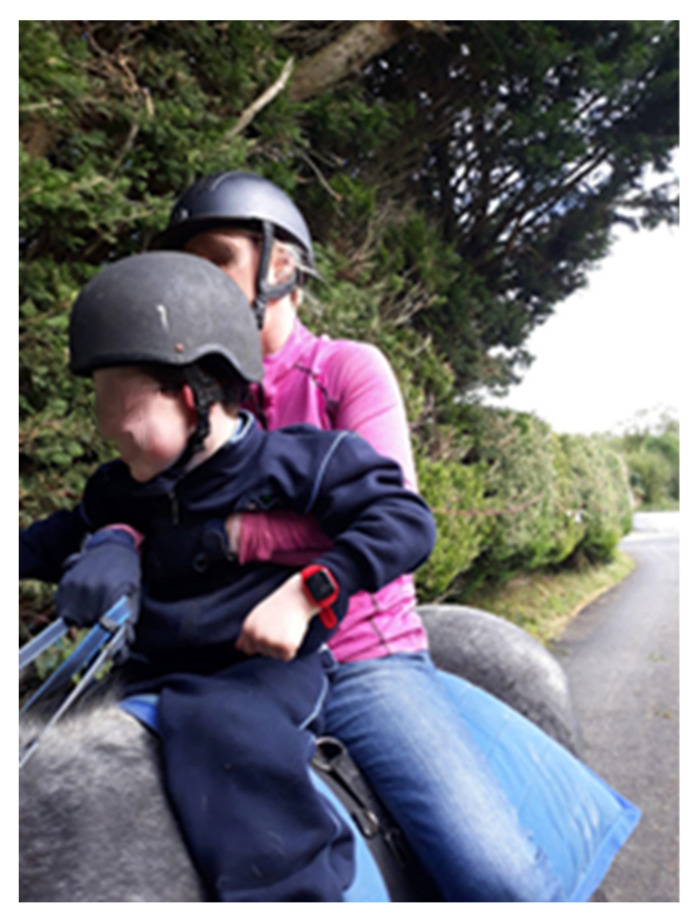
Ethan at his equine therapy session.

Ethan attended the initial focus group with his mother, Cathy. Cathy attended the second quantitative session but was unable to stay for the focus group. Cathy did complete an individual interview in which she elaborated on specific aspects of Ethan’s experience with the watch. Ethan’s family provided six narrated photographs for discussion at the second focus group. Cathy explained that the family choose the photographs because they depicted specific aspects of Ethan’s daily life that they had hoped the Octopus watch would improve. At the initial focus group, she detailed Ethan’s anxious disposition and that the family were eager for the watch to help with alleviating some of this anxiety. Of the six photographs provided, five recorded aspects of Ethan’s life in which the Octopus watch had a positive impact. These aspects included: eating breakfast, equine therapy, sports group and going to school. Cathy explained that Ethan does not have “as many tears in the mornings” since using the watch. In Figure 6, he was happy to go to equine therapy once he received a notification on his watch, where he previously had difficulty with the initiation of this activity. Ethan’s family also provided a photograph of Ethan brushing his teeth but explained that due to sensory issues the Octopus watch did not help as much with this activity.

### 3.3. Themes Identified

**Figure 7:**
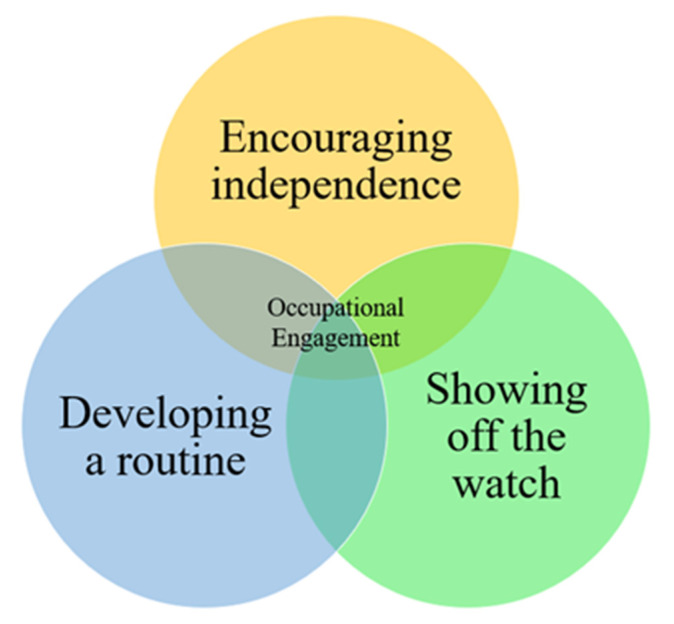
Venn diagram illustrating the overlap between the three identified themes to produce occupational engagement.

Three primary themes emerged from the data: developing a routine, encouraging independence and showing off the watch (Figure 7). These three themes combine to create occupational engagement, a term described by Bejerholm and Eklund as “the extent to which a person has a balanced rhythm of activity and rest, a variety and range of meaningful occupations and routines” [72].

#### 3.3.1. Developing a Routine

At the first focus group, parents expressed hope that the Octopus watch would help with implementing a successful routine into the lives of the children. Overall, it was evident that the watch positively impacted the development of routine for all participants. This theme further divides into two sub-themes: (a) morning routine, and (b) night-time routine.

Three participants reported that the Octopus watch impacted positively on their morning routine, the outlier being Alice. David displayed strong improvement in this area, with his mother Ellen explaining that after three weeks he no longer needed to use the timer function they had been using for breakfast.

*“Before, David was up and down and had no concentration at breakfast-time…now David sits and eats. He doesn’t need to use the timer anymore”.*

After learning to recognise the icons on the watch, Rachel also made progress with developing a morning routine of her own. While previously she relied on instruction from her family, she now uses the Octopus watch to prompt her for tasks such as retrieving her breakfast bowl from the cupboard and making sure she has her bag ready for school. Claire explained that this is a great help to her in the mornings. Cathy also explained that while Ethan still requires physical assistance, the Octopus watch has helped with his anxiety in the mornings and he “has been going on his bus much easier since he has the watch and getting the bus icon”.

**Figure 8:**
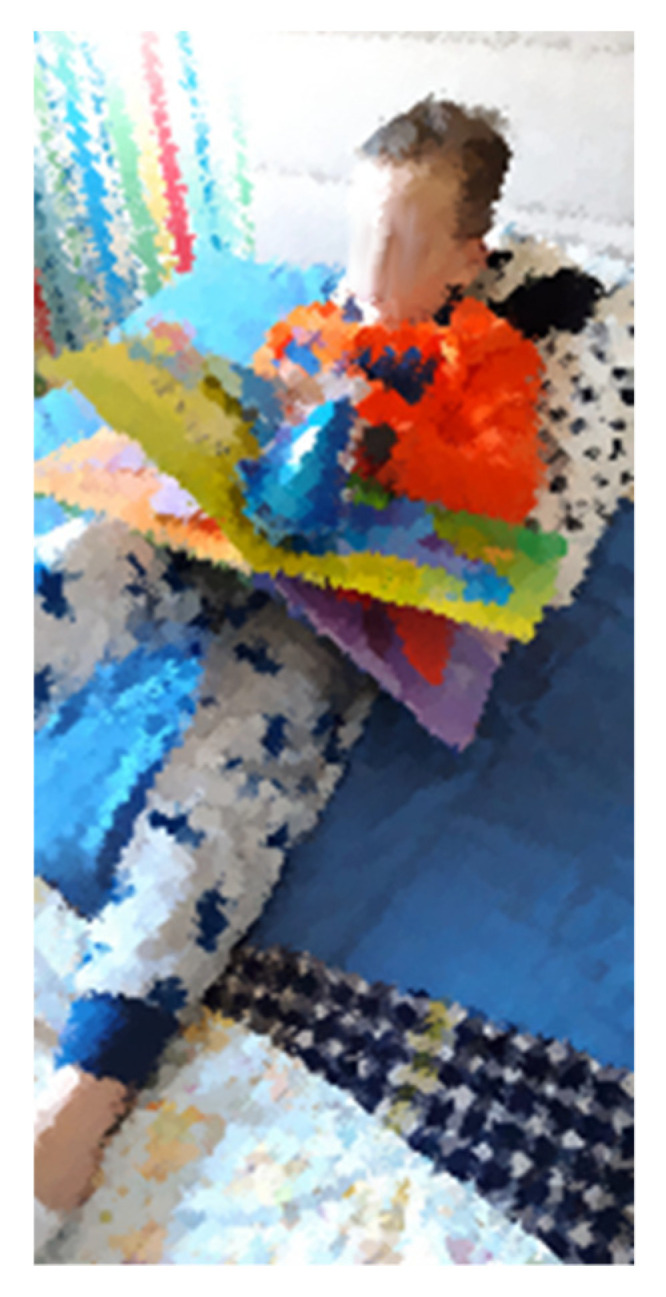
David ready for bed.

Both Rachel and David incorporated reading a story-book into their night-time routine. Both showed improvement in this task. By the end of the third week, Ellen explained, each night David changed into his pyjamas and independently retrieved a book from the shelf (see Figure 8). Similarly, instead of Claire having to initiate the task, Rachel now chooses her own book. Part of Alice’s night-time routine included charging the watch. Anna explained that Alice is determined to place the watch on the charging port herself before going to bed, displaying autonomy.

#### 3.3.2. Encouraging Independence

A big success of the project was that three of the four participants reported an increase in their child’s independence while wearing the Octopus watch. David’s improvement is evident in each of the photographs. Ellen explained that by the end of the third week, David no longer needed the prompts from his watch in the morning as he had already completed his tasks.

*“The timer would go off and David would have task completed. He asks why it is going off if he had done task already”.*

She also explained that David felt safer when wearing his watch as Ellen had enabled the in case of emergency (ICE) function to include both hers and her husband’s phone numbers.

*“Daniel got lost one day about 2 years ago, he was crying, he was very upset. Now he tells me that he can go up to somebody and tell them to ring mammy or daddy and show them the watch”.*

**Figure 9:**
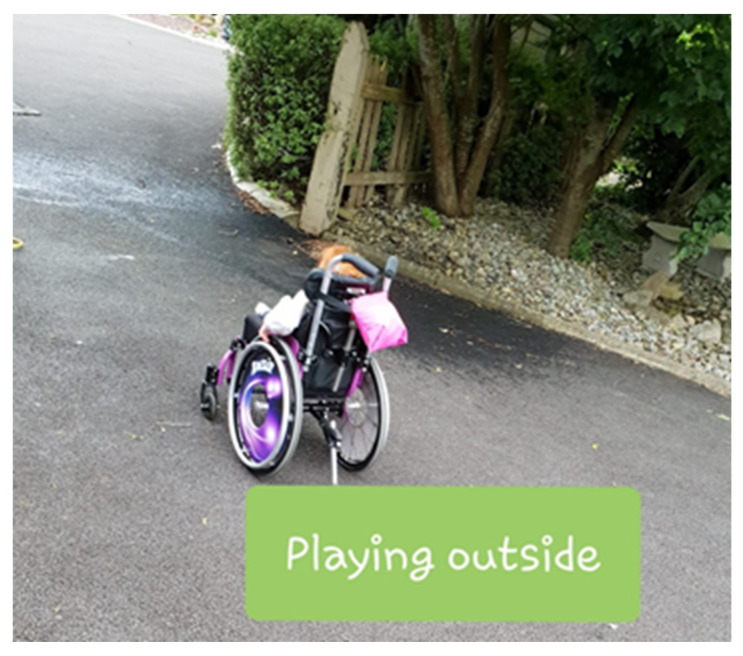
Rachel playing outside.

Claire expressed in the initial focus group that she was eager for Rachel to “be more independent” and her progress is evident through the photographs she provided. Claire expressed her joy at Rachel’s progress with spending time outside after dinner, an activity that Rachel previously did not like to partake in (see Figure 9). She explained;

“At the start, there was a lot of coaxing to go out and I would have to go with her or she wouldn’t stay out for long but by the end of the third week…she would look for me to open the door and she would go out herself—not for long but we are building it every day. I’m delighted to see her achieve this”.

#### 3.3.3. Showing Off the Watch

**Figure 10:**
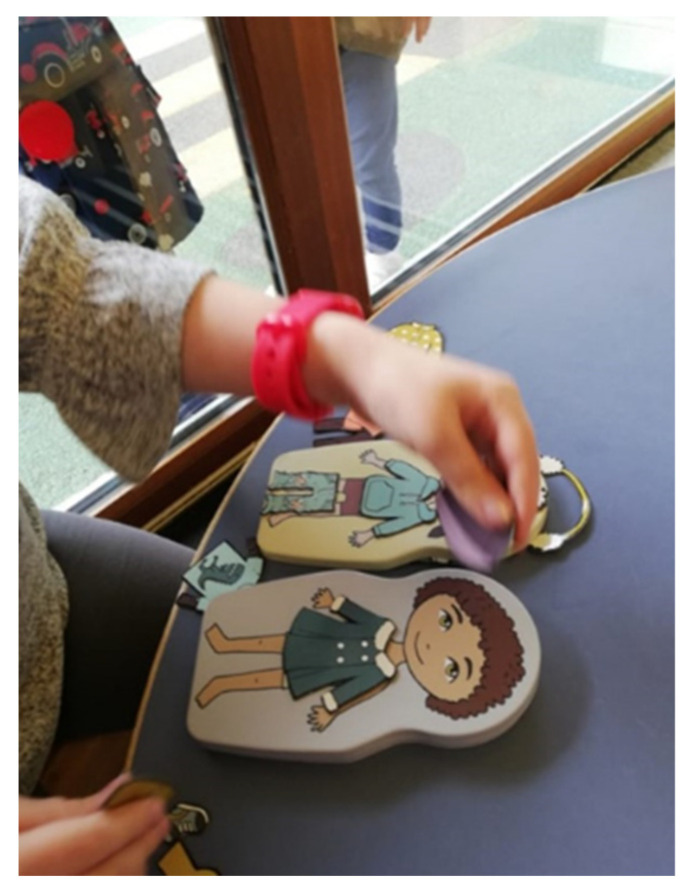
Alice showing her watch to friends at playschool.

Two of the participants reported that their child was eager to show their watch to their friends. During the discussion at the second focus group, Anna explained that Alice had previously distanced herself at a birthday party and usually preferred to be alone rather than join a group. Since wearing the watch, Anna has noticed that Alice has become more sociable at play-school and likes showing her watch off to her friends, depicted in Figure 10.

*“This one was her going into play school in the morning and wanting to show the boys her watch” (in reference to Picture 3 in Supplementary Materials)*

Cathy reported that Ethan also was eager to show his watch to his friends at his sports club.

*“Ethan was excited to go back to his sports group…after the summer break and show his friends his watch”.*

## 4. Discussion

The purpose of this study was to explore whether the Octopus watch is feasible as an early intervention to encourage purposeful ADLs in a group of children (<8 years) living with SB&/H. The great heterogeneity in the needs of children living with SB&/H makes it challenging to form an effective intervention that fosters healthy behaviour. However, the novel Octopus watch, tailored to meet individual children’s needs provides an appropriate platform to encourage purposeful activity. Our mixed-methods findings show promising preliminary evidence for the feasibility of the Octopus watch. Children exhibited improved inhibitory control, increased independence, and routine development in purposeful ADLs. In addition, the process of introducing the Octopus watch provides a method of technology transfer to promote effective use and avoidance of abandonment. Encouraging outcomes were also reflected in user acceptance, with the families responding positively to the intervention. Some evidence was found in terms of increased PA for participants. This is a critical first step, considering the dearth of early intervention evidence for promoting healthy lifestyles in children living with SB&/H.

Firstly, from the CHEXI assessment, supporting interviews and photovoice narratives, the participants appeared to compensate for executive dysfunction. Inhibitory control improved across all participants, although working memory did not change. From previous research, it could have been anticipated that working memory would not improve because no specific memory training was conducted [18]. A noteworthy finding was that two parents noticed that it took a few days for the children to gain an understanding of the icon prompts. This finding suggests that participants needed some time to integrate the new technology into their everyday lives potentially in consequence of the working memory aspect of EF [73]. Improvements in inhibition indicate that the watch compensated for executive dysfunction by facilitating better self-control and reducing impulsively. Diamond states this inhibitory control generates the possibility of choice and change, meaning an increased likelihood of behaviour change and improved ability to choose how to react [74]. For example, in this study, photovoice narratives and semi-structured interviews highlighted less ‘crying, tantrums and screaming’ during the school morning routine, suggesting enhanced self-control. Stubberud et al. reported similar findings with compensatory goal-management training showing that the ability to structure intentions and plan activities significantly improved inhibitory control [12]. Considering that inhibitory control is pivotal in explaining goal-directed behaviour and behavioural flexibility within one’s environment [75], this finding suggests that the icon prompts favourably impacted ADL performance.

Secondly, the satisfaction domain on the COPM showed some evidence of an increase, in contrast to the focus group and interviews where parents described positive ADL performance effects. This discrepancy between quantitative and qualitative findings is possibly due to the small sample size, restricting our ability to judge significance [76]. However, Carswell et al. show that a difference of two or higher on either performance or satisfaction domains is considered clinically significant [55]. Thus, with mean changes of 2.1 and 2.4 on the performance and satisfaction domains, the Octopus watch positively affects purposeful activities.

From the COPM assessment, supporting content analysis and photovoice narratives, the key findings were that the Octopus watch increased independence and supported routine. The results suggest that, possibly due to its user-centred nature with icon prompts promoting ADL goals, the Octopus watch enables the child to lead the intervention [3,77]. Similarly, studies have demonstrated that if the child is involved in the intervention, engagement certainly heightens [15]. Engagement is associated with meaning and meaningfulness and is essential to enhance the value that one assigns to activities [78]. Likewise, the icon prompts appear to increase the value assigned to ADLs, which encourages occupational engagement [79]. Also, occupational engagement could be an explanation for zero technology abandonment witnessed within this study. However, there could have been a novelty period and the intervention phase may have been too short for technology abandonment. Besides client-centredness, another factor that may have contributed to promoting purposeful ADL activity may be determinants outside of the child. From previous studies, parents of children living with SB tended towards overprotectiveness [80,81] because of worry or fear of stigmatizing their child [26]. However, overprotected children often are dependent on adults for guidance and may not have enough decision-making autonomy [82]. Therefore, because parents controlled the daily schedule, their worry may have reduced, thus increasing the child’s independence and participation. The results also indicate that improved participation in purposeful ADLs could positively impact both self-efficacy and quality of life [18], though these factors were not explicitly measured. Van der Slot et al. noted that participation enables self-efficacy [83]. Hammel indicates that the ability to orchestrate ADLs increases an individual’s sense of control and is fundamental to experience an enhanced quality of life [78]. This potential result is consistent with the social cognitive learning theory [84], because the watch icons prompted goal-directed behaviour, overcoming ADLs that were typically avoided, thus increasing the child’s confidence in their ability to maintain occupational engagement in daily routine.

Thirdly, accelerometer data show that David had a significant increase in PA from baseline to intervention, supported with a large effect size. Hence, the Octopus watch benefited this participant’s PA considerably. Alice displayed a mean decrease in PA output from baseline to intervention. One explanation was that the vibration notification function on the watch did not work for this participant, thus hindering intervention benefits. In contrast, both Rachel and Ethan increased mean PA from baseline to intervention. However, these changes were not statistically significant [46]. Power in n-of-1 designs can be increased by longer data collection; in this study, baseline was only four days. Furthermore, qualitative findings suggest that physical limitations were a barrier for both these participants. Rachel and Ethan were the only two participants who used a wheelchair full-time with their average level of PA considerably less than David’s (independently ambulant) and Alice’s (part-time ambulant). Peny-Dahlstrand et al. found that non-ambulatory children with SB had significantly less activity compared to ambulatory children with SB [85]. Moreover, despite the insignificant PA change, the mean increase in PA could still have been beneficial for both children.

Besides personal barriers, it is feasible that our 16-day intervention was too short to elicit significant changes in the complex task of increasing PA for each participant. Supporting this explanation, in the O’Brien et al. review the shortest randomised controlled trial (RCT) PA intervention for children who mobilise with wheelchairs was three months [86]. Similarly, another review analysing school-based PA interventions in typically developing children found that most effective interventions were over one year [87]. Whether a longer intervention could produce more significant PA changes in children living with SB&/H needs to be further examined. Of note, PA interventions for children living with SB&/H are in their infancy, with limited translational research displaying the effectiveness. Furthermore, our study contributes preliminary transitional evidence, that the Octopus watch is beneficial for increasing PA for three of four children studied here. This is a noteworthy finding, considering the sedentary behaviour of SB&/H children reported in the literature [16], and the potential benefits of PA improving health [88].

Fourthly, consistent with Puri et al. [31], technology acceptance of the Octopus watch was measured primarily by one question: do you think this watch would be sustainable/feasible over a longer period? All parents responded positively to this question with one parent stating; “it’s only going to make things better”. Key smartwatch components which appeared to contribute to this technology acceptance include: aesthetics, real-time feedback rewards, and the element of play. Some parents viewed the Octopus watch as a fashion icon in school, with Mercer et al. reporting that aesthetics is a primary reason for device acceptance [89]. In addition, the real-time feedback rewards on the watch after completion of ADLs emerged as a powerful feature, providing a motivational component. These findings are in line with the STARFISH interactive application study, where real-time feedback benefited behaviour change [20]. Further, in the present study, the reward system on the Octopus watch appeared to embody the key aspect of ‘play’ [90]. For instance, one play-based activity was beating the watch timer before it finishes to achieve full stars. Such play-based activities with the Octopus watch seemed to increase the child’s willingness to engage in purposeful ADLs, consequently encouraging better routines and habits. Thus, our findings suggest that the Octopus watch is acceptable and feasible for the children living with SB&/H. Though, assistive technology transfer was not immediate for all participants, from photovoice narratives and semi-structured interviews the Octopus watch was successfully integrated into each of the children’s lives over the course of the study with parents reporting effective use. This is an important finding as it suggests that introducing the Octopus watch with clear information incorporated with set goals increases the sustainability and user success for children and families living with SB&/H.

Finally, the Octopus watch is one helpful component for strengthening routines and independence which may contribute to family resilience. Family resilience is generally understood as the capacity of the family to remain positive when faced with a disruptive situation affecting their stability and integrity. Resilience enables the family to come out stronger [91]. Holmbeck and Devine found that parents of children living with SB&/H experience increased psychological, physical and social demands compared to parents of typically developing children [92]. Despite these demands, the Octopus watch seemed to embed the intervention in each family routine which could increase family satisfaction. Thus, overall, the user-centred nature and repetitive icon feedback seemed to enhance purposeful ADLs, fostering family resilience through occupational engagement.

### 4.1. Limitations

Although this case series used the participants as their own controls [46], the small sample size and non-randomised nature of the study design means that the positive observed effects could be due to the intervention, but also may be a reflection of non-specific intervention effects (e.g., researcher attention/exaggerated reporting) or even a combination. Therefore, this study precludes any definitive causal interpretation and generalisability [61]. In addition, due to technical issues, PA data was lost for unknown reasons, questioning the reliability of the watch in activity measurement. Finally, one researcher was somewhat new to conducting semi-structured interviews which may have influenced the answers given. However, this mixed-method pilot study provides preliminary evidence for the effectiveness of the Octopus watch and warrants a larger-scale study to address the noted limitations.

### 4.2. Future Research and Recommendations

A reliability study should be conducted on the Octopus watch with a subsequent RCT to support more conclusive findings. To enable generalisability, a large sample size is recommended, thus, facilitating a power analysis before data collection is necessary [65]. Other considerations include a longer intervention period, with a follow-up phase. In addition, further research may explore the potential benefits of the Octopus watch with other groups who experience executive dysfunction, for example, children living with autism spectrum disorder, attention deficit hyperactivity disorder or cerebral palsy who experience impairment of executive functioning.

## 5. Conclusions

In conclusion, the Octopus watch appears to be a feasible early intervention for children living with SB&/H and is a promising user-friendly smartwatch that places the user’s ADL goals at the centre, thus enabling the child to increase independence and autonomy through icon prompts. The consistent goal-based icon repetition of the smartwatch acted as a compensatory strategy for EF and thus ameliorated impaired inhibition which appeared to yield increased purposeful ADL participation. Further, all participants adopted the smartwatch and showed potential to increase PA. Aspects which contributed to technology acceptance were aesthetics, real-time feedback rewards and play-based activities. Overall, the increase in purposeful ADLs and evident technology acceptance strengthened family resilience by fostering occupational engagement. This is a substantial finding as sustainability assistive technology use for children living with SB&/H was questionable due to associated difficulties with goal-directed skills. This study is a small but noteworthy step toward finding a translational early intervention that is beneficial to promoting better routines and habits for children living with SB&/H. Hence, the findings provide direction for further large-scale studies.

## Figures and Tables

**Table 1:** Developmental milestones (adapted from Goracke et al. [29]).

Developmental Milestone	Typically Developing Infant	Infant with SB
Sitting	6–7 months	1–2 years
Crawling	7–11 months	1–2 years
Standing	9–13 months	3 years
Walking	12–15 months	3–7 years

**Table 2:** Inclusion criteria for level of functional mobility based on Hoffer et al. [47] and Schoenmaker’s [48] ambulation level classification, with an additional functional wheelchair component.

Level of Functional Mobility	Description
Normal ambulation	Independent and unrestricted ambulation without use of assisted devices.
Community ambulation	Independent outdoor ambulation with/without use of brace and/or assisted devices; using wheelchair for longer distances.
Household ambulation	Using braces or assisted devices for indoor ambulation; using wheelchair for outdoor locomotion.
Non-functional ambulation	Walking only in therapeutic situations.
Functional wheelchair user	Wheelchair for mobility but can self-propel the wheelchair.

**Table 3:** Participant demographics.

	Age	Sex	Ethnicity	Assistive Devices	Independently Walking
Rachel	4	F	Irish	Wheelchair	No
David	7	M	Irish	None	Yes
Alice	3.5	F	Irish	Braces (full-time) Wheelchair (Part-time)	Yes (short distance)
Ethan	7	M	Irish	Wheelchair (full-time) Braces (full-time)	No

SB and H = spina bifida and hydrocephalous, M = male, F = Female.

**Table 4:** N-of-1 analyses comparing baseline and intervention PA.

	Physical Activity (Octo-Points)
**Rachel**	
*r* ^2^	0.003
*p*-value	0.833
**David**	
*r* ^2^	0.28
*p*-value	0.021 *
**Alice**	
*r* ^2^	0.042
*p*-value	0.397
**Ethan**	
*r* ^2^	0.004
*p*-value	0.796

Note: * *p* < 0.05.

**Table 5:** Childhood executive functioning inventory (CHEXI) individual change scores from pre-test to post-test.

	Pre-Test	Post-Test	Difference	% Change
**Rachel**				
Working memory	41	40	−1	−2.4
Inhibition	35	29	−6	−17.1
**David**				
Working memory	46	45	−1	−2.2
Inhibition	43	37	−6	−14
**Alice**				
Working memory	38	41	3	7.9
Inhibition	49	37	−12	−24.5
**Ethan**				
Working memory	44	41	−3	−6.8
Inhibition	50	45	−5	−10

CHEXI = childhood executive functioning inventory.

**Table 6:** Comparison of Canadian occupational performance measure (COPM) individual scores at pre-test and post-test intervention with corresponding participants goals.

	Pre-Test	Post-Test	Mean Difference	Individual Goals
**Rachel**				Feeding/mealtime Washing hands Brushing teeth Independence Outside play
Performance	3.8	5.4	1.6	
Satisfaction	4.4	5.8	1.4	
**David**				Toileting Storytime Homework Medication Morning routine
Performance	3.2	7.6	4.4	
Satisfaction	3.4	7.2	3.8	
**Alice**				Feeding/mealtime Daily routine Toileting Clean-up Walking
Performance	5.0	6.6	1.6	
Satisfaction	4.6	7.4	2.8	
**Ethan**				Brushing teeth Medication Toileting Outings (school) Homework
Performance	4.6	5.4	0.8	
Satisfaction	4.4	5.8	1.4	

COPM = Canadian occupational performance measure.

## Supplementary Materials

The following are available online at https://www.mdpi.com/1660-4601/17/22/8316/s1.
