# Supplementary material for: Octopus Watch Fosters Family Resilience by Enhancing Occupational Engagement for Children with Spina Bifida and/or Hydrocephalus: Pilot Study
Source: Int J Environ Res Public Health. 2020 Nov 10;17(22):8316. doi: 10.3390/ijerph17228316 (PMC7697938; doi:10.3390/ijerph17228316)
Supplement: Supplementary file 1 [file ijerph-17-08316-s001.pdf]

International Journal of
Environmental Research and Public Health

MDPI

5. Supplementary

Supplementary 1 - Demographic questionnaire

Demographic Questionnaire

Check the appropriate boxes and fill out the necessary information in the spaces below:

Q. What is your child's age?

Q. What is the sex of your child?

☐ Male

☐ Female

☐ Other

Q. What is your child's ethnicity?

Q. Does your child use assistive devices part-time or full-time, or not at all?

	**Part-time**	**Full-time**	**Not at all**
**Walker**			
**Braces**			
**Wheelchair**			
**crutches**			
**Other?** Please specify below			

Q. Can your child walk independently?

☐ Yes

☐ No

☐ Sometimes

Q. Does your child have Spina Bifida, hydrocephalous or both?

☐ Spina Bifida

☐ Hydrocephalous

☐ Both

Supplementary 2 – Semi-structure interview (Pre-test)

Semi-Structured Interview exploring barriers to activities of daily living (PRE-INTERVENTION)

Q1. What does your child do currently from day to day?

Probe: Self-care, leisure, productivity, play

Q2. What would you like your child to be doing?

Q3. What does your child have difficulty doing during the day?

Probe: What do you think causes these difficulties?

Q4. Is there anything that gets in the way of your child being more physically active?

Probe: Environment, social, individual

Q5. How do you think the Octopus watch could aid/assist your child?

Supplementary 3 - Semi-structure interview (Post-test)

Semi-Structured Interview exploring the octopus watch (POST-INTERVENTION)

Q1. How did your child find using the Octopus watch?

____

Q2. Did you think the watch benefited your child?

Probe: Did the watch help with daily routine? Motivation?

____

Q3. Did you or your child have any difficulty with the Octopus watch?

____

Q4. How would you compare your child's daily activity before the octopus watch to when your child was wearing the octopus watch?

____

Q5. Did your child respond to the watch icons consistently over the two weeks?

If yes: Do you think this watch would be sustainable/feasible over a longer period?

If no: Why you think this happened?

____

Supplementary 4 – Photographs and Narrations

4.1. Rachel

Picture 1: Rachel has pre-school 4 mornings a week and using the schedule I set a reminder about 15 mins before we were due to leave. Rachel learned that once she felt the icon buzz and saw the school icon, she knew she had to go and get her bag.

For the first two weeks she would tell me when the watch buzzed and I would encourage her to get her bag. During the week just passed we had great success in that she would go herself and get her bag and only call me if she was stuck in getting it.

Picture 2: Another good success. I used breakfast time as part of her schedule and she now knows to go to the press and bring out her bowl and give it to me.

I also found the timer of great help and we used to play Beat the Timer!

Repetition is what Roisin works well with and I will continue using the watch.

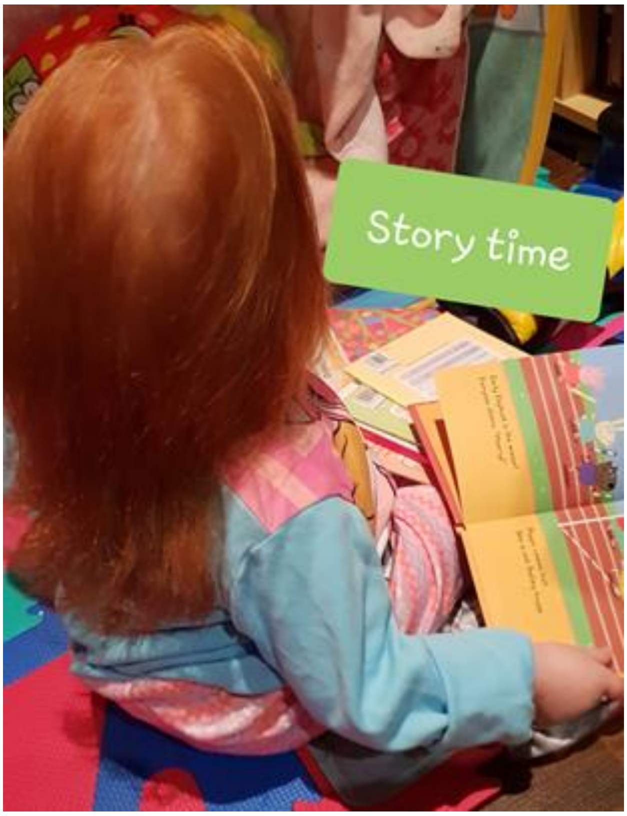

Picture 3: Rachel loves her books and loves her reading – it's part of the routine before bed.

I included this as part of her schedule even though she looks forward to it every night.

Again, starting off she was getting used to the buzz telling her it was story time.

Last couple of evenings she has gone to get her own books. She would call me then to help her take one out for her.

Picture 4: This was one of our great successes. Rachel doesn't like to go outside on her own or sometimes with her siblings. I included this as part of her free time. At the start, there was a lot of coaxing to go out and I would have to go with her or she wouldn't stay out for long but by the end of the third week once she got used to the idea and doing the same thing every day, she would look for me to open the door and she would go out herself – not for long but we are building it every day. I'm delighted to see her achieve this.

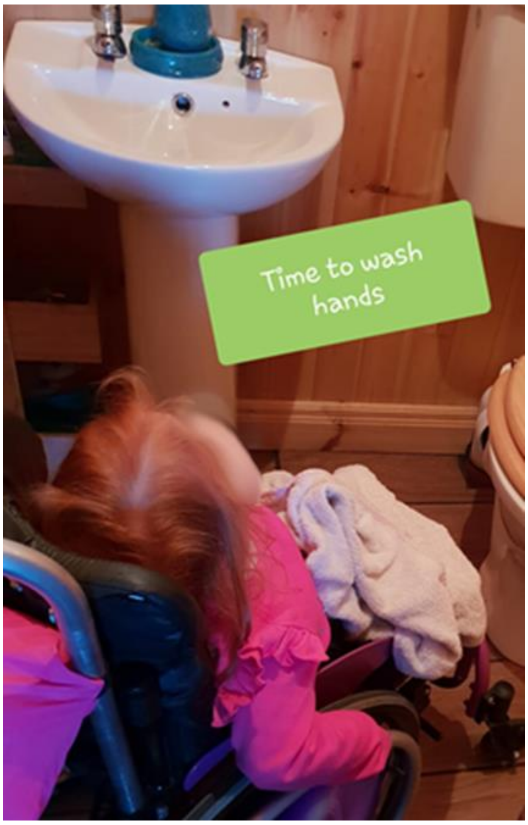

Picture 5: I made washing her hands part of her schedule as she wheels with her wheels instead of her rims and we are constantly washing her hands as they get very dirty.

With this task I have to help her as she is not able to reach the sink but tells me now when to wash hands and if she can, she will go and get her towel.

4.2. David

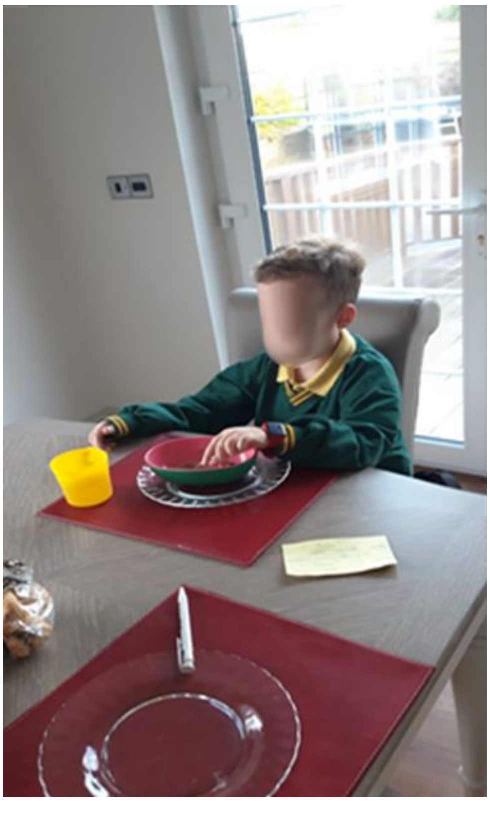

Picture 1: Before, David was up and down, no concentration at breakfast-time. Took a long time. Put on timer for breakfast, 20 mins to eat breakfast.

Now David sits and eats. Doesn't need to use timer now.

Notification for tablet. Tablet left on table. Confirm to mam that is done.

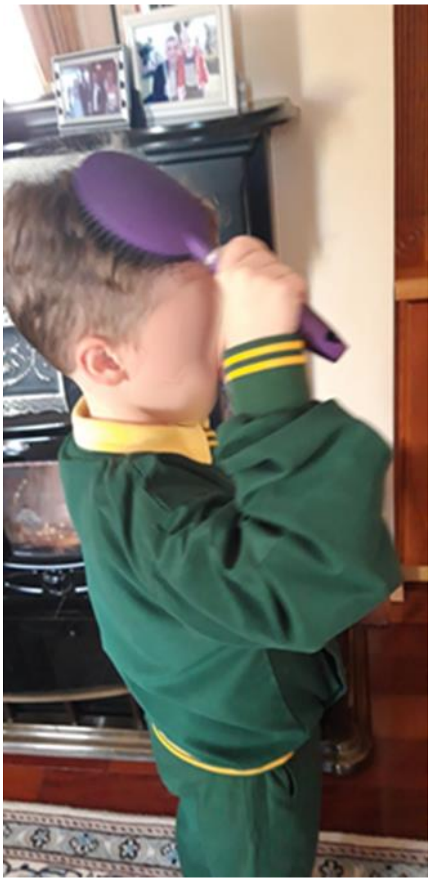

Picture 2: Getting ready for school.

Before, he wouldn't have hair brushed, or ready for school.

Tell mom he had teeth brush, hair done, breakfast ate.

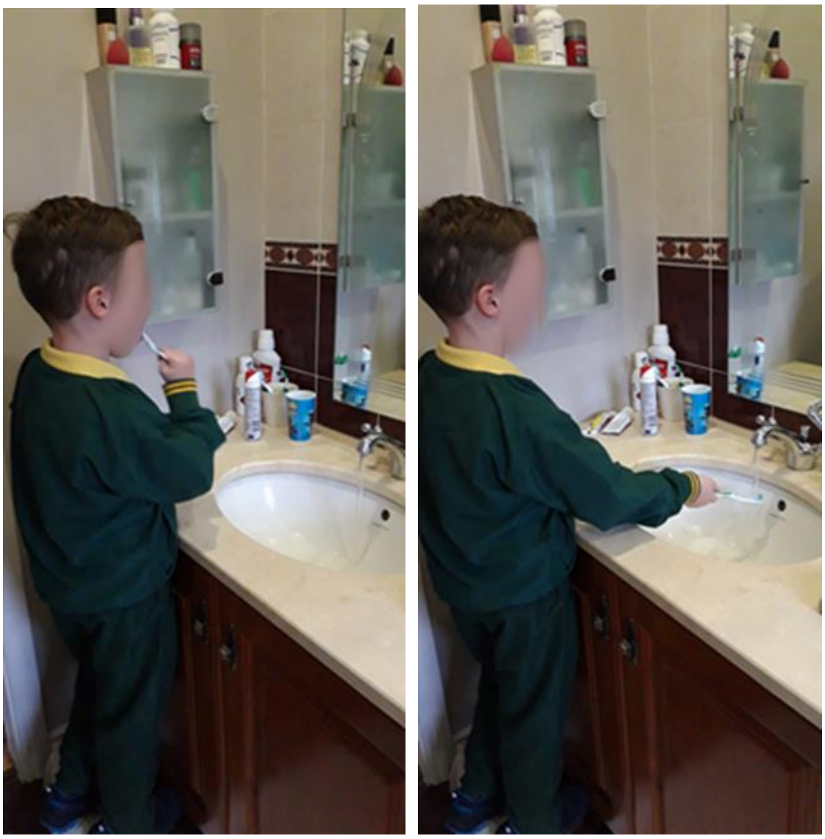

Picture Series 3: Ahead of time for brushing teeth.

The timer would go off and David would have task completed. He asked why it is going off if he had done task already.

Picture Series 4: Happy going to school

No screaming any more

On time for school

Picture 5: Downtime before bed, reading at home.

Before David wouldn't be reading, wouldn't get the book, wouldn't dress himself.

In this photo, David dressed himself and got the book himself. Sits on the bed and reads himself.

Yesterday we went to the library, and got three books out, this was the first time we could do this.

4.3. Ethan

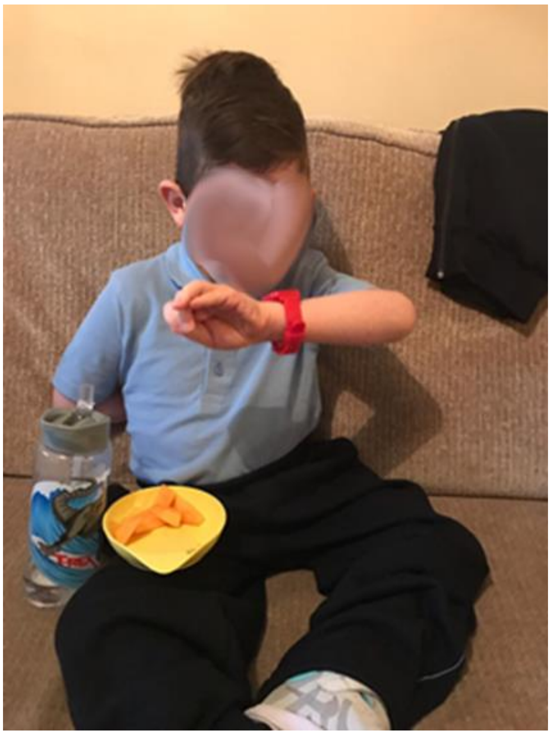

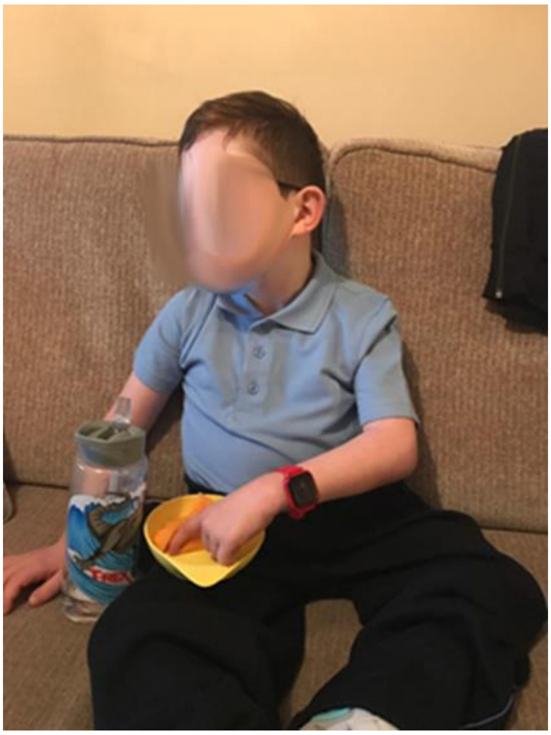

Picture Series 1: Ethan is not a good eater in the mornings. I have always struggled to get breakfast into him before he goes to school but since having the Octopus watch when he sees the breakfast icon, he is happy to have a small bit of breakfast.

Picture 2: Ethan does equine therapy once a week.

He doesn’t always want to do it and there can be tears before he gets up on the horse

But not this week, he got the icon and was happy to get on the horse.

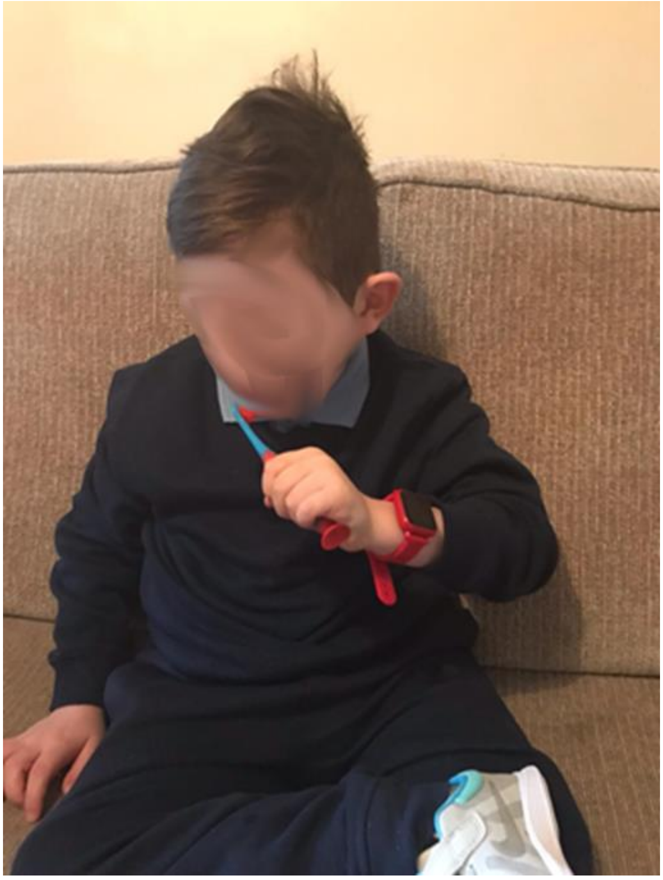

Picture 3: Teeth brushing is a big issue with Ethan due to sensory problems, unfortunately the watch hasn't helped as much in this area.

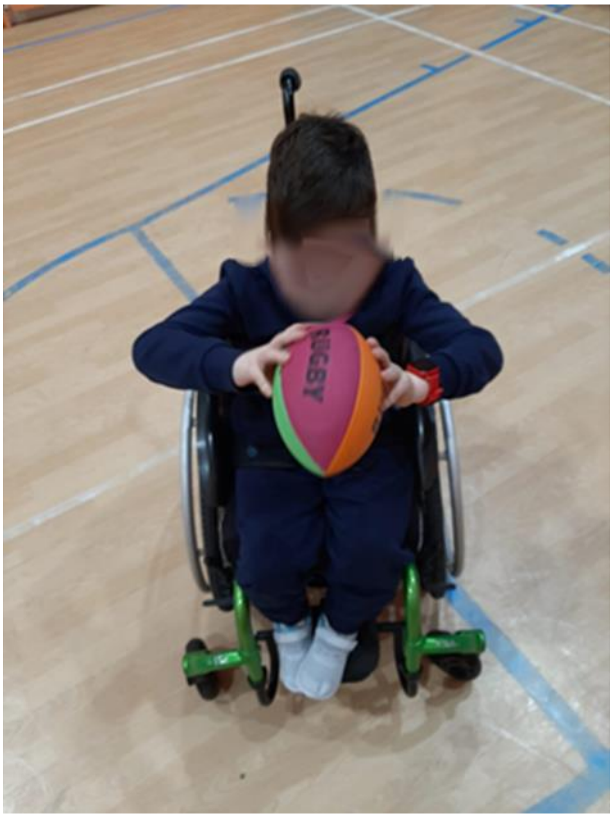

Picture 4: Ethan was excited to go back to his sports group…after the summer break and show his friends his watch.

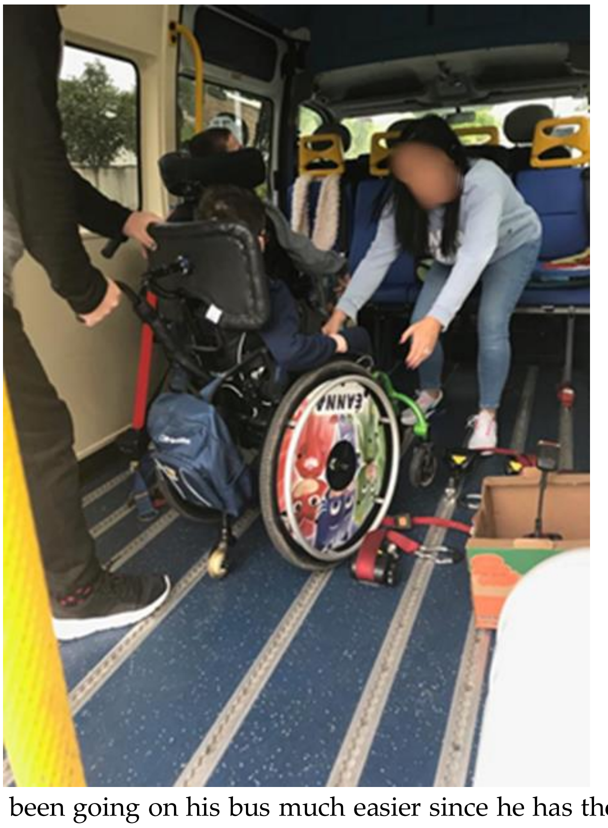

Picture 5: Ethan has been going on his bus much easier since he has the watch and getting the bus icon.

Not as many tears in the mornings.

4.4. Alice

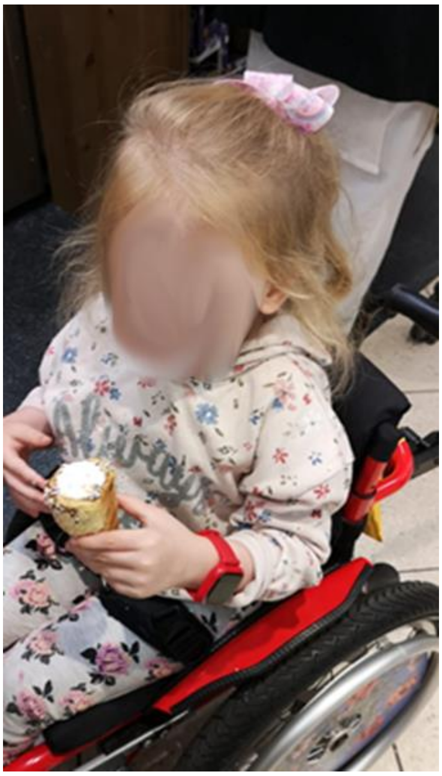

Picture 1: This photograph shows Alice shopping with her mother after play-school. Anna had included this in the scheduler on the octopus app.

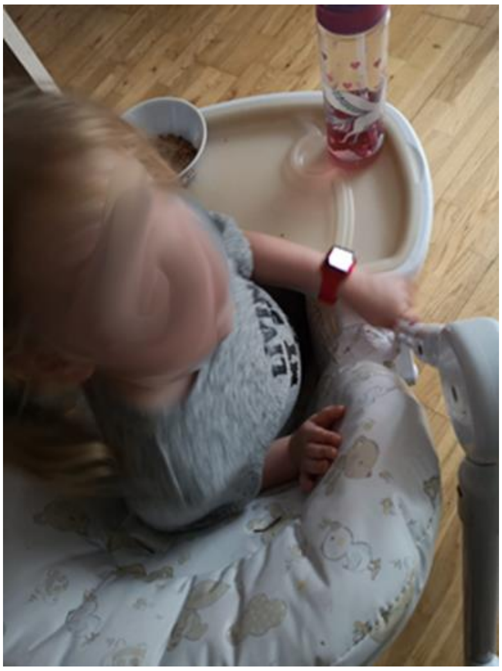

Picture 2: In this picture, Alice's watch is signifying that it is now time for 'Free Play'. Instilling the routine of free play after dinner-time has encouraged Alice to eat more and finish her dinner on time.

Picture 3: Wearing the octopus watch has improved Alice's social skills in play-school. She loves to show her watch to the children in her class.

Picture 4: Alice has experienced increased autonomy since receiving her octopus watch. Each night she ensures that the watch is placed on the charging port by herself.

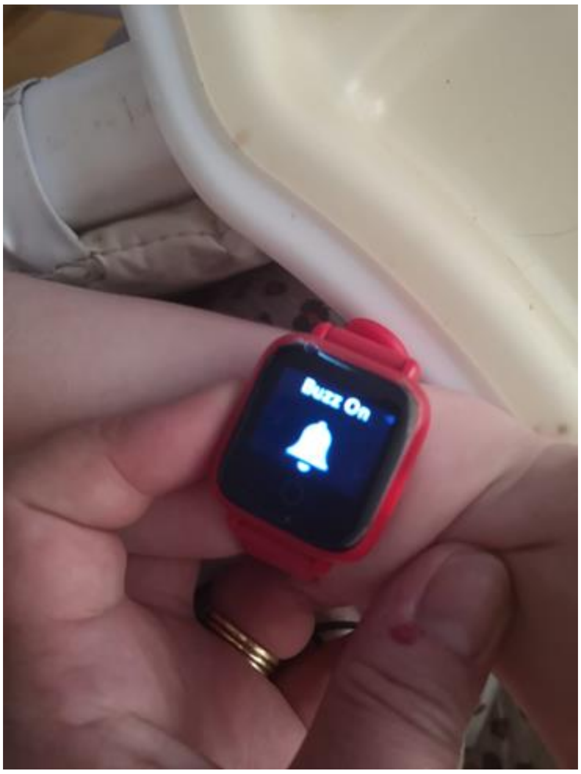

Picture 5: This picture depicts that the vibrate function was enabled on Alice's watch although it was reported to have malfunctioned during the study period.

Supplementary 5 – Accessible Information Package (AIP)

5.1. Octopus watch poster

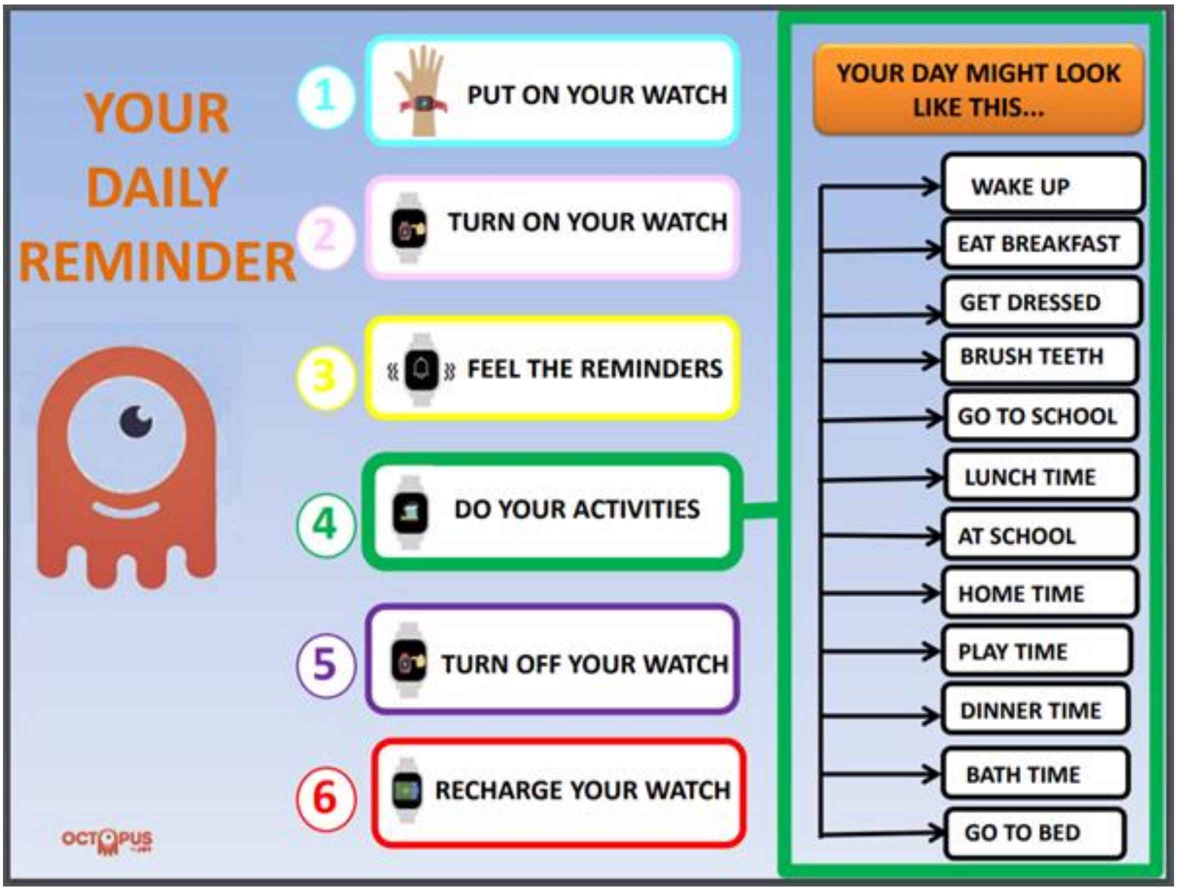

5.2. Octopus watch Quick Start Guide

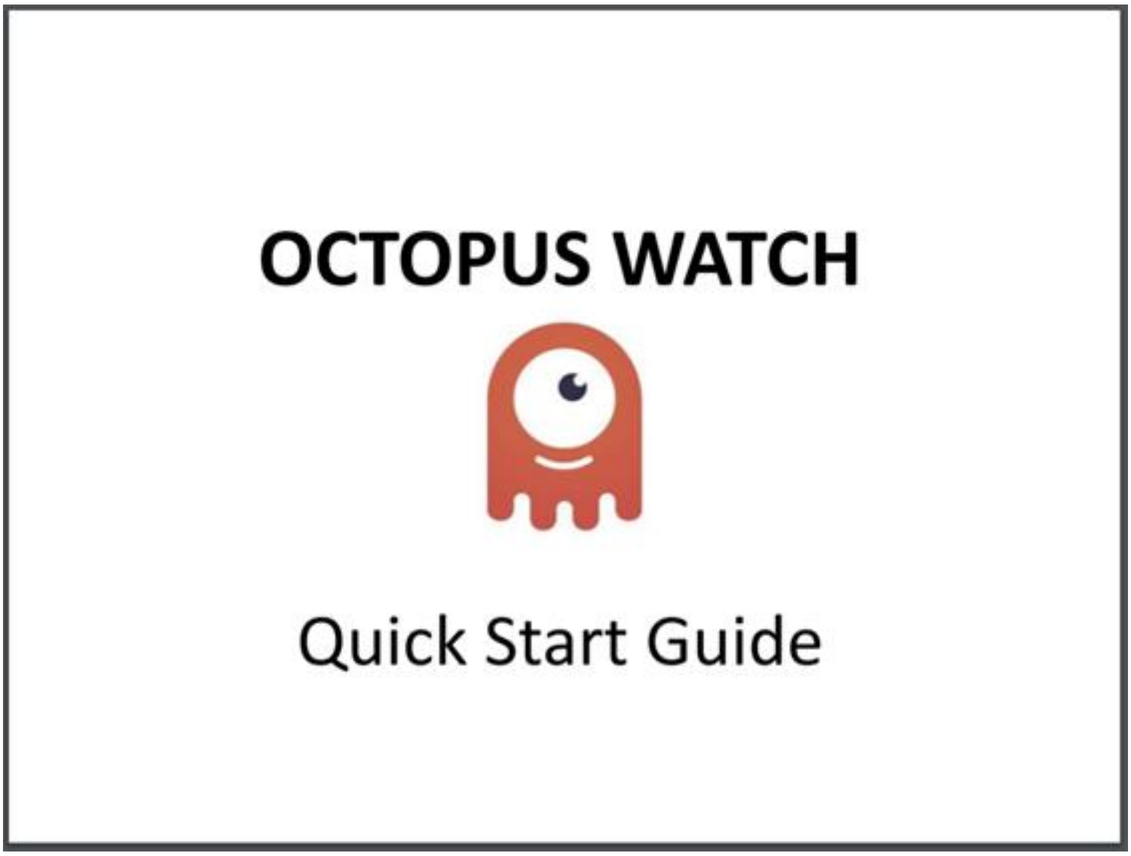

WELCOME

The Octopus Watch is the first icon based watch that empowers children by teaching good habits and the concept of time, while also encouraging them to stay active. It is a watch, a scheduler, and an assistant. The watch is based on the power of suggestion and helps your child manage their day and their expectations of what comes next.

This guide will help you get started.

Enjoy!

TABLE OF CONTENTS

BEFORE YOU BEGIN, YOU NEED TO 4

NAVIGATION 5

CHARGE THE WATCH 6

SIGN UP FOR AN OCTOPUS ACCOUNT 7

VALIDATE YOUR ACCOUNT 8

PAIR YOUR WATCH AND PHONE 9

CREATE A REGULAR SCHEDULE (ANDROID) 10

ADD AN EVENT TO THE SCHEDULE 11

ACTIVATE MOTION TRACKER 12

SYNCHRONIZE YOUR WATCH 13

FUNCTIONS 14

PRINTING GUIDELINES 15

MORE INFORMATION 16

BEFORE YOU BEGIN, YOU NEED TO…

1. Install the free Octopus app on your iPhone 9) or Android phone (5.1).

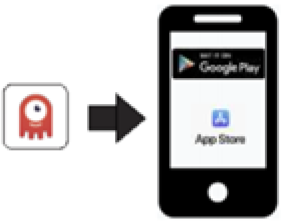

2. Go to **Settings** > **Bluetooth** and enable **Bluetooth.**

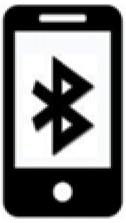

3. Turn on WiFi.

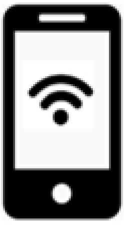

NAVIGATION

FRONT

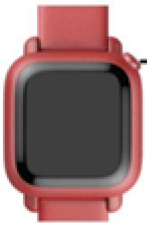

Multifunction button

Power On/Off

Menu

BACK

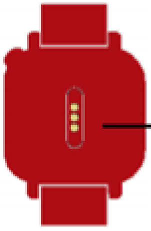

Charging Port

CHARGE THE WATCH

Before you charge your Octopus watch, ensure that the charging port is clean.

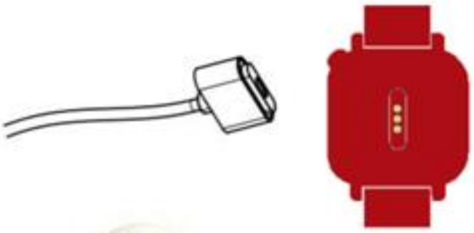

- Attach the USB charging cable to the magnets located at the back of the watch and plug the cable in.

OR

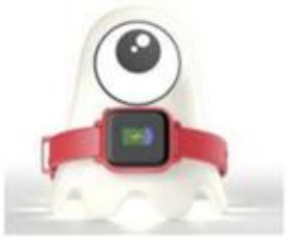

- Attach the watch to its companion and plug the companion in.

The watch takes approximately 1 hour to fully charge. You can view the battery power level on the watch face.

SIGN UP FOR AN OCTOPUS ACCOUNT

1. On your phone, open the Octopus app .

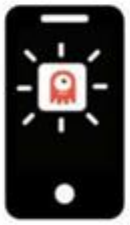

2. Enter your personal details and click **Next**.
   You are brought to the Octopus home screen.

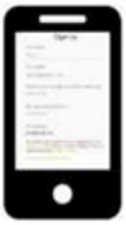

VALIDATE YOUR ACCOUNT

1. Connect to your email account.

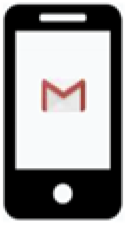

2. Open the email from support@heyjoy.io and click **Confirm my account**.

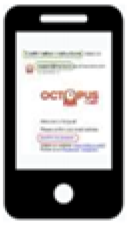

3. On the **Login** page, sign into your Octopus account.

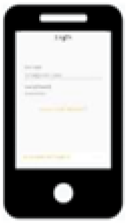

If you do not confirm your email within 3 days, you will be locked out of your account.

PAIR YOUR WATCH AND PHONE

1. Turn on both your watch and your phone. Ensure Bluetooth is enabled.

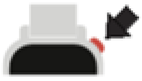

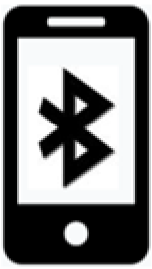

2. On your phone, open the Octopus app and click **Add a watch**. A message telling you the phone has been found is displayed.

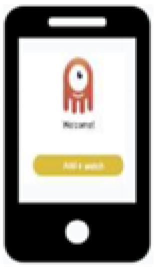

3. Click **Connect** and enter the name of the child who will wear the watch.

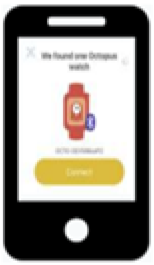

4. Click **Next.**

CREATE A REGULAR SCHEDULE (ANDROID)

1. Open the **Kids** menu by clicking the top right corner.

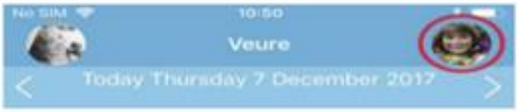

2. Click the icon and select **New Blank Schedule**.

3. Select **Regular**.

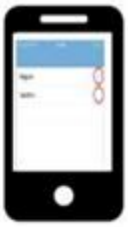

ADD AN EVENT TO THE SCHEDULE

1. Click the icon.

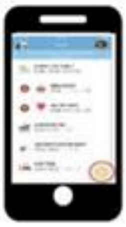

2. Search for the desired icon.

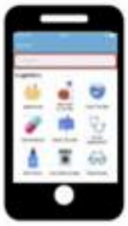

3. Customize and save the event.

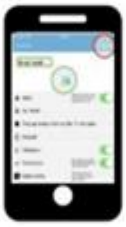

ACTIVATE MOTION TRACKER

1. In the Octopus app, go to the right hand menu, and tap **Motion**. 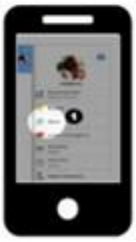
2. Tap **Edit**. 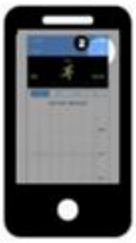
3. Enable motion tracking. 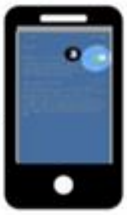
4. Synchronize the watch.

SYNCHRONIZE YOUR WATCH

Ensure your watch is up to date with the latest firmware version(right hand menu > **Firmware Update**)

1. Enable Bluetooth and location on your phone. 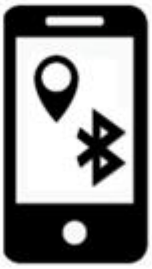
2. Click 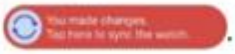.

3. Activate Bluetooth on the Octopus watch.
   The synchronization initiates automatically. 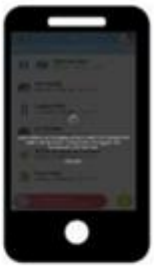

FUNCTIONS

Double press the multifunction button to enter the Octopus Watch's menu screen. Once you are in the menu, press the button to cycle through each feature.

Power On/Off	Turn on/off your watch (double press to turn off).
In Case of Emergency (I.C.E)	Add parental contact and/or important medical information.
Timer	Set a timer for 1, 3, 5, 10, or 15 minutes (double press to enter).
Battery	View battery level.
Clock	View the time and set the alarm.
Buzz	Turn all vibrations on or off (double tap to turn on and off).
Info	View useful information about your watch.
Build	View manufacturer information about your watch .

PRINTING GUIDELINES

We recommend printing this document using the following settings:

Page Size & Handling
- Booklet
- Booklet subset: Both sides
- Binding Left

Orientation
- Portrait

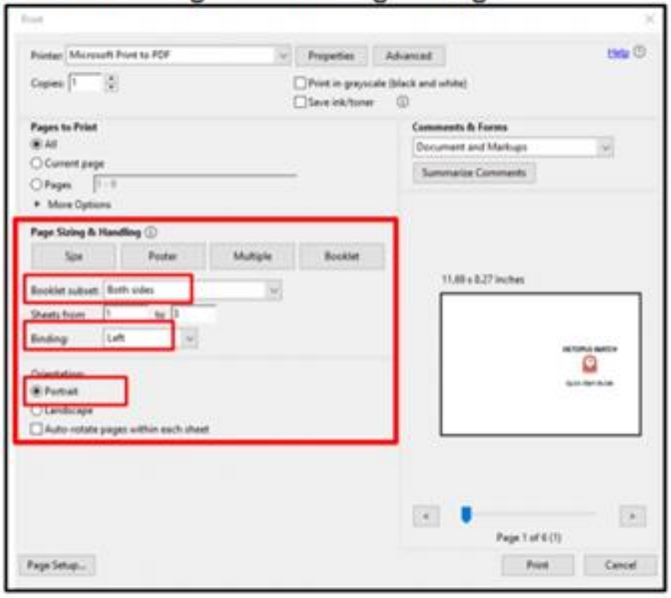

We also recommend binding this booklet using a spiral binding method.

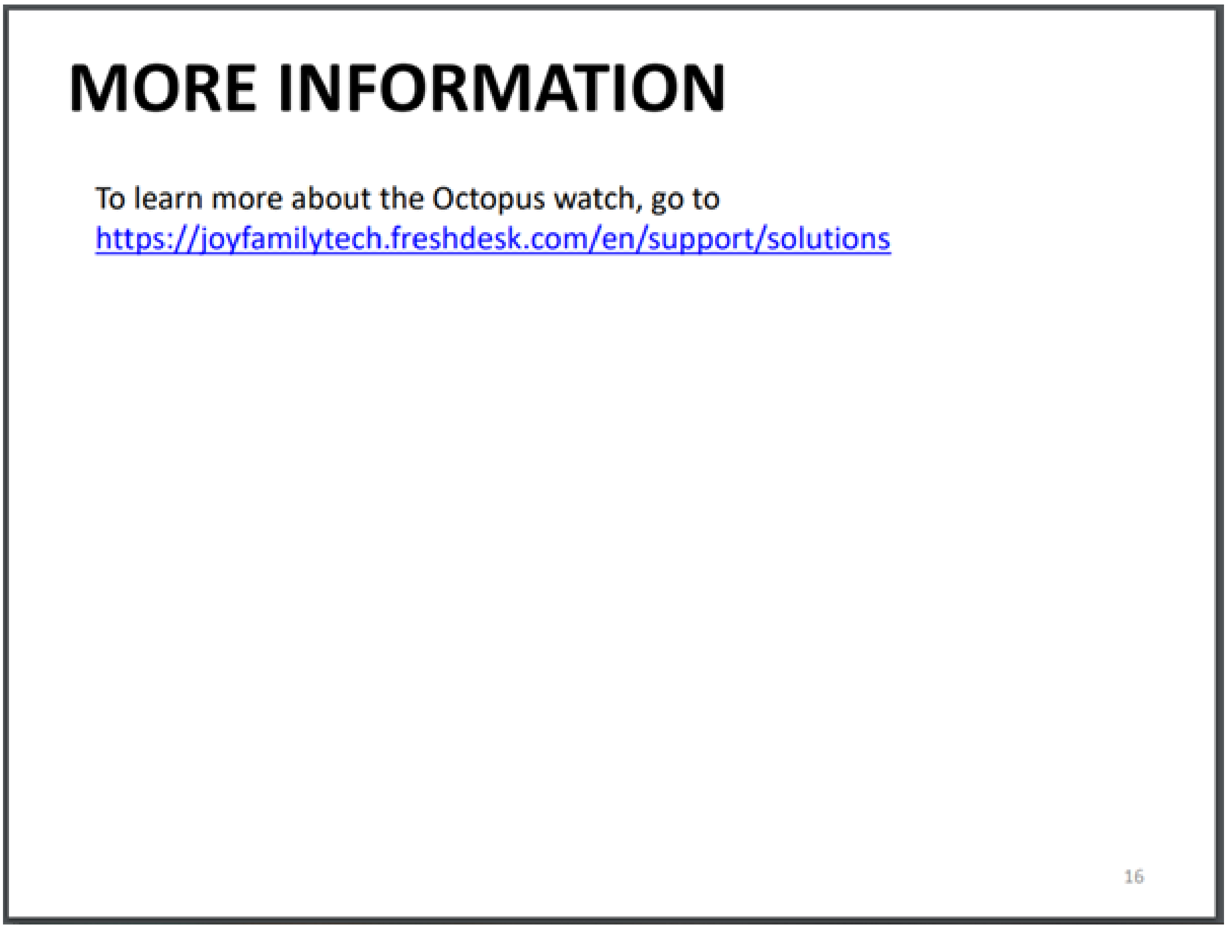

Supplementary 6 – COPM and Semi-structured interview content analysis

<u>*Research aim 1.*</u> To investigate the watches potential in promoting PA. The primary theme which surfaced from the qualitative data regarding physical activity was; '*Physical limitation as a barrier*'. Three of the four participants identified physical function as a barrier to improvements in PA during the intervention phase. This barrier appeared to emerge in consequence of ambulation level which restricted activity improvements:

> *I wouldn't say it made a difference with physical activity. Yeah, because he can only do what he can do, and the watch wasn't going to change that for him.* [Ethan]
>
> *She is quite stuck anyway, she needs a lot of things done for her.* [Rachel]

<u>*Research aim 2.*</u> To examine if the watch compensates for EF deficits. The primary theme which developed from the qualitative data regarding EF was; '*Sense of control*'. Improvements in the inhibition domain appeared to exhibit increased control over each participant's behaviour, attention, emotions or thoughts. As the parents voiced below, the watch noticeably enhanced the participants' ability to overcome poor habits or strong impulses to do what's more appropriate:

> *It wasn't screaming get your coat, get your shoes on, and everything wasn't ready in the morning. Mornings where just nuts like in the house, but I will say he was on time and a happier child going to school, definitely.* [David]
>
> *But definitely did improve things. With the morning routine and getting ready for school and getting out the door without tantrums and crying.* [Ethan]

It is harder to do the homework when we get home. The teacher was suggesting just writing one word rather than writing one sentence. But he didn't want to be different and now he times it, we give it thirty minutes for the homework and if it is not done in thirty minutes, we close all the books. [David]

Research aim 3. To explore the potential effects of the intervention on purposeful ADL's every participant created five client-centred goals based on the COPM assessment. The primary theme which emerged from the qualitative findings was; '*Independence and routine support*'. The significance of the octopus watches as an assistive technology appears to both support and promote independence, surfacing throughout all four participants. Participants Alice and Ethan voiced that the icon prompts were beneficial as they assisted independence through breaking down the tasks into smaller steps:

It gave him independence. I will say he used to prompt me and that you know. Like mommy, I have to eat now. [David]

It was definitely prompting her on what to do. I think the prompts are definitely the most beneficial because it's breaking it down into smaller steps, and it's giving her independence. [Alice]

It's breaking it down, it's giving her independence and it's breaking down the instructions. [Ethan]

Routine was supported through the timer function for two of the four participants, specifically improving time management, with David voicing the importance of completing tasks. The watch icons seemed to assist as an external motivator for the participants which yielded and increased the willingness to engage in routine for all participants, as echoed by the below quotes:

It definitely was a motivator for him. To do different things and to get it done! He was constantly on the timer. The timer was used for breakfast, for getting ready and if he had to read the book, the timer. Going to bed at night, the timer. [David]

Like eating his breakfast in the morning, it's always a struggle to get breakfast into him, he doesn't like eating breakfast especially. He gets collected very early in the mornings to go to school, so, when he sees his breakfast icon, he just seems a little bit more willing to eat a small bit of breakfast. [Ethan]

Participant Rachel and David also describe a noteworthy improvement in independence, prompting participants to seek new activities. David initiated going to the library after refusing to go for the past year. Whereas, Rachel previously did not go outside alone, however, is now successfully going outside in the back garden:

We haven't gone to the library for the whole year. Yesterday he asked me to go to the library and picked three books out of the library and he sits now with his book at night-time and he flicks the pages himself. So, I am delighted with that, that is huge! [Ethan]

We were saying about how she didn't like going outside on her own, even with the kids. But she's made great success with it! I used it as the free time in the schedule. In the evening time, she would go out. Now not for long, but she would go out on her own. [Rachel]

Research aim 4. To explore user experience of the watch in terms of usability and acceptability, the primary theme which developed from the qualitative data was; '*Technology acceptance strengthened family resilience*'. Across all participants, less resistance to routine was apparent due to technology acceptance and adherence. Furthermore, throughout each participant, the octopus watch appears to

strengthen family resilience through promoting routine, family satisfaction, independence, sense of security and incorporating play-based activities:

I think it's only going to improve things. I definitely, one hundred percent I think it's going to make things better! Because from the few little prompts she was seeing, you could see the change. So, I am really excited to see it! [Alice]

But definitely did improve things. Definitely with the morning routine and getting ready for school and getting out the door without tantrums and crying. [Ethan]

He loved the idea with the mammy and daddy's number on there. Because he got lost once, he didn't really get lost, but he got lost once in Tesco, he got so upset and frightened by the whole thing. So, he said mammy if I ever get lost now, I can just go up and tell someone to ring one of those numbers. [David]

It is harder to do the homework when we get home. The teacher was suggesting just writing one word rather than writing one sentence. But actually, he didn't want to be different and now he times it, we give it thirty minutes for the homework and if it is not done in thirty minutes, we close all the books. [David]

Both participant one and two incorporated the key element 'Play' and used it in a competitive and light-hearted fashion below adopting seemingly a play-based learning approach. Moreover, the power of the octopus watches and play appeared to foster family collaboration, providing an opportunity for the children to build better habits and routine:

She used the timer an awful lot and we used to play this game beat the timer, and that worked really well for us. [Rachel]

He was up earlier in the morning, the teeth were brushed, the hair was done then, he was down for breakfast. So, he could be down at breakfast and it said brush my hair, but he said, mammy, I have that done. So, he used to be trying somewhat to get ahead of the watch to beat it! He was delighted then if he was ahead, so he knew that he was doing everything right! [David]
